# Power-Free Sweat Sample Concentration Using a Silica-Gel-Packed PDMS Microchannel

**DOI:** 10.3390/polym18020260

**Published:** 2026-01-18

**Authors:** Hirotada Hirama, Masanori Hayase

**Affiliations:** 1 Integrated Research Center for Self-Care Technology, National Institute of Advanced Industrial Science and Technology, Chiba 277-0882, Japan; 2 Faculty of Science and Technology, Tokyo University of Science, Chiba 278-8510, Japan

**Keywords:** microfluidics, sample concentration, wearable biosensing

## Abstract

In recent years, diagnostic technologies that utilize noninvasively collected sweat have garnered significant interest. However, the concentration of components in sweat is lower than that in blood, making the introduction of a concentration step as a sample pretreatment crucial for achieving highly sensitive detection. In this study, we developed a PDMS-based microchannel filled with silica gel, a desiccant particle, to concentrate liquid samples at room temperature without requiring an external power source or heating. The evaluation of the basic characteristics of the fabricated microchannel confirmed that filling it with silica gel efficiently removed the solvent vapor from the liquid samples. In concentration tests using the fluorescent dye uranine as a model for sweat sugar, a maximum 1.4-fold concentration was achieved in DPBS solution and a 1.2-fold concentration in artificial sweat at room temperature. In contrast, no similar concentration effect was observed in microchannels without silica gel packing. The proposed silica-gel-packed PDMS microchannel features a simple structure and requires no external equipment, making it easily integrable with existing microfluidic devices as a sample pretreatment module. This method is considered useful as a passive and simple sample concentration technique for the analysis of low-molecular-weight components in sweat.

## 1. Introduction

Histological diagnosis, which involves surgically removing tissue from the body and performing diagnostics based on that tissue, is highly invasive and places a significant burden on patients [1]. Against this backdrop, liquid biopsy, a diagnostic technology that utilizes bodily fluids that can be collected minimally invasively or non-invasively, has garnered significant attention in recent years [2,3]. Liquid biopsy is a diagnostic technology that analyzes biomarkers (substances reflecting the presence or progression of a disease) in bodily fluids, such as sweat, urine, saliva, and blood [4]. Among bodily fluids, sweat and saliva have gained attention as targets for liquid biopsy analysis because they can be collected less invasively than blood samples [5]. Sweat can be continuously collected from the skin surface and contains diverse low-molecular-weight components that reflect physiological and metabolic status.

Microchannels are formed on silicon, glass, and resin substrates using microfabrication techniques. Bioanalysis and chemical reactions are performed by flowing reagents and samples through the microchannels. Microfluidics offers advantages such as precise fluid control under laminar flow conditions, reduced reagent consumption, and shorter reaction times [6,7]. Consequently, applying microfluidics to liquid biopsies is expected to reduce the diagnostic time and decrease the required sample and reagent volumes [3]. In recent years, liquid biopsy research using microfluidics has been actively conducted [8]. Polydimethylsiloxane (PDMS) is widely used in these devices because of its high biocompatibility, low cytotoxicity, gas permeability, and ease of processing [6].

Sekine et al. developed a skin-adhesive microfluidic device capable of in situ analysis of electrolytes in sweat, demonstrating the quantification of sweat components during exercise via fluorescence intensity measurements [9]. Furthermore, Gao et al. reported a wearable sensor system capable of simultaneously analyzing multiple metabolites and electrolytes in sweat, demonstrating the utility of sweat analysis for noninvasive diagnosis and physiological monitoring [10]. Furthermore, Xiao et al. reported a wearable microfluidic device capable of detecting sugars in sweat via colorimetry, which is anticipated to be a noninvasive method for monitoring blood glucose levels in patients with diabetes mellitus [11]. However, the sugar concentrations in sweat are significantly lower than those in blood, making highly sensitive detection difficult without modification [12]. Consequently, the choice of usable detection reagents and methods for sweat sugar analysis using wearable microfluidics is limited, and achieving highly sensitive and accurate measurements remains challenging [11]. In recent years, significant progress has been made in wearable and microfluidic sweat analysis, including evaporation-driven microfluidic patches, capillary-based sweat collection systems, and low-cost passive sweat collection devices [13,14,15,16,17,18,19,20]. Several recent studies published between 2019 and 2024 have demonstrated continuous sweat transport and sensing using evaporation-enhanced or capillary-driven designs, highlighting the rapid development in this field. These approaches enable continuous sweat transport and sensing without active pumping, significantly advancing noninvasive physiological monitoring. However, when sample preconcentration is required to enhance the detection sensitivity, many reported systems rely on elevated temperatures, external gas flow, or integrated heaters to accelerate solvent evaporation. These requirements may limit their applicability in fully power-free, disposable, or long-term wearable systems, particularly when simple integration and low system complexity are desired features. Therefore, a simple and passive sample pretreatment strategy that enables sweat sample concentration at room temperature without external energy input is required. Introducing a pretreatment process to concentrate sample components prior to the detection step is an effective approach to address this challenge.

Numerous microchannels have been reported for concentrating components in liquid samples. Xu et al. developed a microchannel that utilized the properties of PDMS to evaporate the solvent by heating the liquid sample, thereby concentrating its components [21]. Although this method offers the advantage of a very simple structure, making it suitable for integration with analytical microchannels, the requirement for heating during concentration imposes limitations on its application to heat-sensitive samples and its deployment in wearable applications that do not require an external power source, such as batteries. The efficiency of sample concentration based on solvent removal strongly depends on the moisture absorption characteristics of the desiccant, particularly the adsorption rate and the saturated moisture adsorption capacity. A higher adsorption rate enables the rapid removal of solvent vapor, which is especially important under continuous flow conditions, whereas a sufficient adsorption capacity is required to sustain the concentration over extended operation times. Silica gel exhibits a moderate moisture adsorption rate and well-defined saturation behavior under ambient conditions, allowing stable and controllable solvent removal without abrupt changes in flow or channel blockage. Compared with other commonly used drying agents, molecular sieves possess a higher moisture adsorption capacity and stronger affinity for water; however, they typically require high-temperature regeneration and may induce excessive drying or flow instability in confined microfluidic structures. Calcium chloride is inexpensive and highly hygroscopic; however, its tendency to deliquesce can lead to liquid formation and potential clogging in microchannels. In contrast, silica gel offers a favorable balance between adsorption kinetics, capacity, chemical stability, and safety. Although this comparison is qualitative, it highlights the practical trade-offs among commonly used desiccants in the context of passive, power-free microfluidic concentration systems for wearable applications. Therefore, in this study, we fabricated a PDMS-based microchannel filled with silica gel, a desiccant particle, to concentrate liquid samples without requiring an external power source or heating by adsorbing and removing the solvent vapor within the microchannel. We evaluated the basic characteristics of the microchannel and demonstrated the feasibility of sample concentration using a fluorescent dye as a model for sugar in the sweat samples.

## 2. Materials and Methods

### 2.1. Fabrication of Microchannels

**Figure 1:**
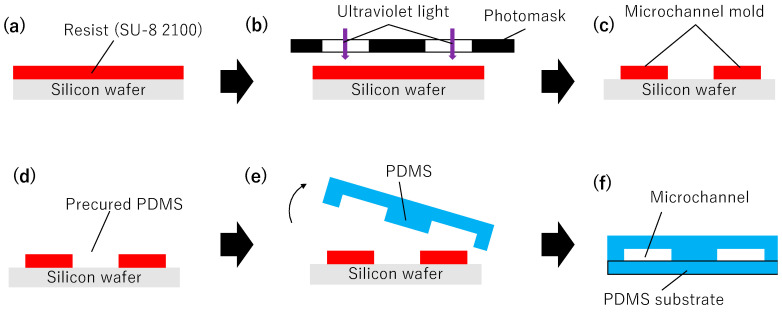
Fabrication procedure of a PDMS microchannel used for sweat sample concentration by soft lithography. (**a**) Spin coating of photoresist on a silicon wafer. (**b**) UV exposure through a photomask. (**c**) Development of the photoresist to form a microchannel mold. (**d**) Casting of precured PDMS on the mold. (**e**) Demolding of the patterned PDMS slab. (**f**) Bonding of the PDMS slab to a PDMS substrate by O_2_ plasma treatment.

PDMS-based microchannels were fabricated using conventional photolithography [6,22] (Figure 1) and filled with silica gel desiccant particles. Silica gel was selected as the desiccant for this study because of its low cost, chemical stability, and extensive knowledge regarding its moisture absorption properties.

#### 2.1.1. Fabrication of Microchannel Molds

To fabricate the microchannel molds, SU-8 2100 (Kayaku Advanced Materials, Westborough, MA, USA) was uniformly spin-coated onto a 4-inch silicon wafer (film thickness 130 μm) (Figure 1a), soft-baked (6 min at 65 °C, 40 min at 95 °C), exposed using a mask aligner through a photomask with the desired channel pattern (Figure 1b), post-baked (5 min at 65 °C, 14 min at 95 °C, and again 1 min at 65 °C), immersed in SU-8 developer for 16 min, and finally rinsed with isopropyl alcohol (Figure 1c). The UV exposure was performed using a mask aligner (PEM-800, Union Optical Co., Ltd., Tokyo, Japan) with an exposure dose of 15 mJ cm^−2^.

#### 2.1.2. Fabrication of PDMS Microchannels

The main and curing agents of PDMS (SILPOT 184, Toray Dow Corning, Tokyo, Japan) were mixed well in a 10:1 ratio and thoroughly degassed under reduced pressure. PDMS was poured onto the microchannel mold and heated in an oven at 80 °C for 2 h (Figure 1d). The PDMS curing was conducted in an oven (SONW-300SB, AS ONE Corporation, Osaka, Japan) at a temperature fluctuation of ±3 °C. After heating, the microchannel mold was peeled off the cured PDMS, and a PDMS plate with a patterned microchannel was obtained (Figure 1e). Finally, the PDMS sheet with the patterned microchannels was surface-treated using oxygen (O2) plasma (treatment conditions: 100 W, 30 s) for hydrophilization. This sheet was then laminated with another PDMS sheet that had also been surface-treated with O2 plasma and bonded by heating (80 °C, 2 h) (Figure 1f). A plasma reactor (PR510, Yamato Scientific Co., Ltd., Tokyo, Japan) was used for the O_2_ plasma treatment.

#### 2.1.3. Filling the PDMS Microchannel with Silica Gel

A microchannel filled with silica gel was fabricated using the following procedure. Silica gel was sieved to select particles between 45 and 90 μm. The microchannel was designed with a PDMS pillar spacing of 40 μm to allow gas and liquid passage while preventing silica gel passage. Utilizing this structure, a silica gel slurry was injected from the Inlet (P), and the dispersion medium was withdrawn from the Outlet (L) using a syringe to fill the silica gel. Ethanol and silicone oil were evaluated as dispersion media for the silica gel. Silica gel suspended in volatile dispersion media was injected into the microchannel (P) via the inlet (P). Minor external vibrations were applied to ensure uniform filling and complete filling of the PDMS microchannel with silica gel without gaps. Subsequently, to completely remove the dispersant from the PDMS microchannel, the PDMS microchannel was placed in a 120 °C oven for 10 min and then cooled to room temperature.

### 2.2. Sample Concentration Test

**Figure 2:**
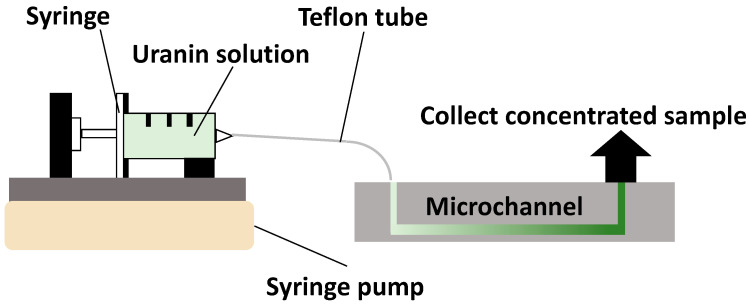
Schematic illustration of the experimental setup for the sample concentration experiments.

Uranine (FUJIFILM Wako Pure Chemical Corporation, Osaka, Japan) was used as a sugar model for the sweat. Uranine was selected because it is water-soluble, chemically stable, and allows for highly sensitive optical quantification. Although uranine is not a sugar, its molecular weight and diffusion behavior are comparable to those of glucose in aqueous media. In this study, artificial sweat (Hayashi Pure Chemical Ind., Ltd., Osaka, Japan) was used to control the sample composition and ensure reproducibility. A syringe was connected to the inlet (L) of the silica gel-filled microchannel using a Teflon tube. A syringe pump (KDS100, KD Scientific, Holliston, MA, USA) was used to deliver DPBS (Life Technologies Japan Ltd., Tokyo, Japan)/uranine solution (uranine concentration: 5 μg/mL) and artificial sweat/uranine solution (uranine concentration: 5 μg/mL) to the microchannel at a constant flow rate (Figure 2). A calibration curve was prepared to quantify the uranine concentration in the post-concentration solution. Uranine solutions were prepared at concentrations of 0.1, 0.5, 1, 5, and 10 μg/mL in the solvents (DPBS and artificial sweat). The absorbance of the prepared uranium solutions was measured using a spectrophotometer, and a calibration curve relating the uranine solution concentration to its absorbance was constructed. To investigate the effect of residence time on the concentration ratio within the fabricated microchannel, the flow rate was set to 0.01, 0.02, 0.06, 0.12, and 0.24 mL/h. The residence time was calculated by dividing the previously determined effective channel volume by the sample flow rate. Concentration was performed by controlling the flow rate to achieve residence times of 0.5, 1, 2, 5, and 10 min for the liquid sample in the channel. To quantify the uranine concentration in the concentrated solution, the solution recovered from the outlet (L) was measured using a spectrophotometer. For all experiments, a microchannel without silica gel packing was fabricated and used as the control. All experiments were conducted at room temperature (23 ± 2 °C) under ambient laboratory conditions. The relative humidity during the experiments was approximately 40–60%.

## 3. Results and Discussion

### 3.1. Fabricated Microchannels

**Figure 3:**
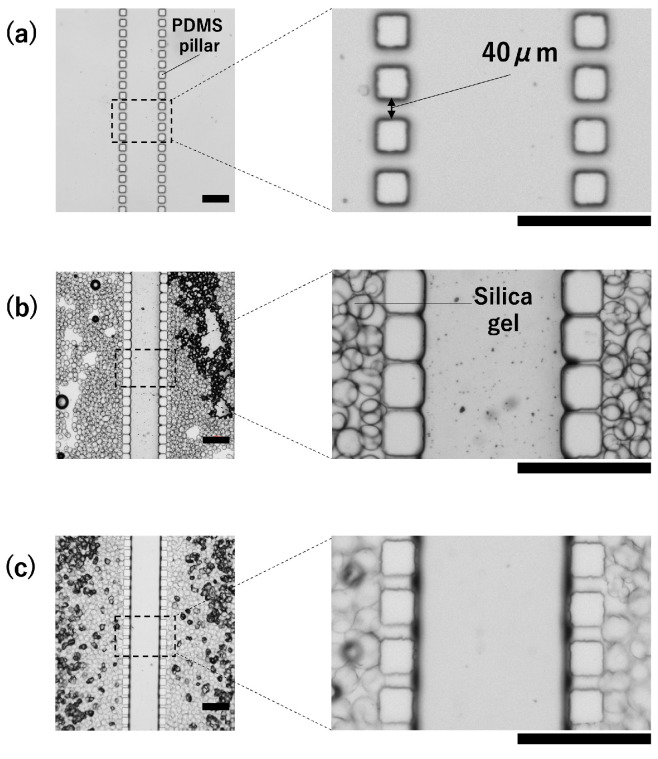
Examination of the dispersion medium of silica gel. Scale bars are 400 μm. (**a**) Microchannels before introducing silica gel dispersion, (**b**) Microchannels after introducing silica gel dispersed in silicone oil. Swollen PDMS pillars were observed. (**c**) Microchannels after introducing silica gel dispersed in ethanol.

**Figure 4:**
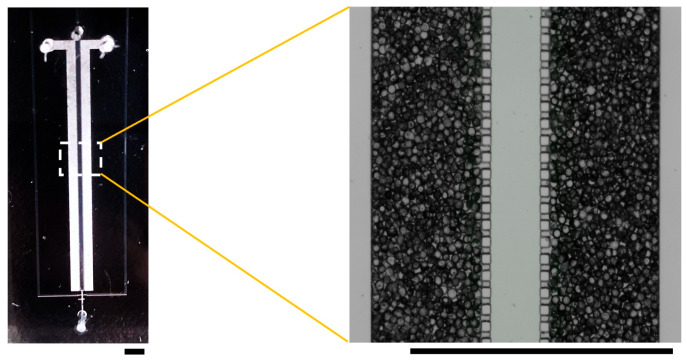
Fabricated microchannel with packed silica gel. Scale bars are 2 mm.

First, we investigated the dispersion media for silica gel (Figure 3). When silicone oil was used as the dispersion medium, the PDMS pillars absorbed silicone oil and swelled, as shown in Figure 3b. PDMS is known to swell in organic solvents and has been reported to have a high affinity for substances such as silicone oil [23]. The swelling of the PDMS pillars prevented the silicone oil delivered from the inlet (P) from passing through the pillars. Instead, it remained trapped inside the microchannel, making it impossible to fill the microchannel with only silica gel. When ethanol was used as the dispersion medium, PDMS did not swell, as shown in Figure 3c, allowing the silica gel to fill the microchannels. Therefore, ethanol was used as the dispersion medium for the silica gel in the subsequent experiments. The use of ethanol as the dispersion medium for silica gel enabled the fabrication of a microchannel uniformly filled with silica gel (Figure 4). Furthermore, as designed, the images confirmed that the silica gel did not leak into the central part of the microchannel (where the liquid sample flowed through).

### 3.2. Evaluation of Basic Microchannel Characteristics

**Figure 5:**
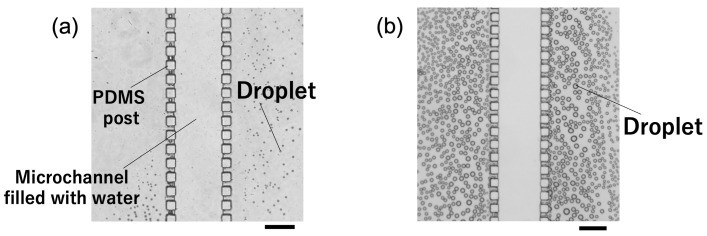
Vapor transportation in microchannel at (**a**) room temperature. (**b**) 80 °C. Scale bars are 300 μm.

PDMS is known to have a high gas and vapor permeability [24]. Based on this characteristic, as a replication of a previous study [21], we observed the trapping of solvent vapor (water vapor in this experiment) by flowing ultrapure water through a microchannel that was not filled with silica gel (Figure 5). At room temperature, water droplets were observed in the microchannel (P) (Figure 5a), likely due to water vapor condensation. In contrast, when the PDMS microchannel was heated to 80 °C, the number of water droplets increased significantly compared to that at room temperature (Figure 5b). This trend is consistent with the results reported in previous studies [21] and is thought to occur because heating promotes water evaporation, increasing the amount of water vapor inside the microchannel. All sample concentration experiments in this study were conducted at room temperature, and observations under heating conditions were performed to confirm the vapor transport mechanism.

**Figure 6:**
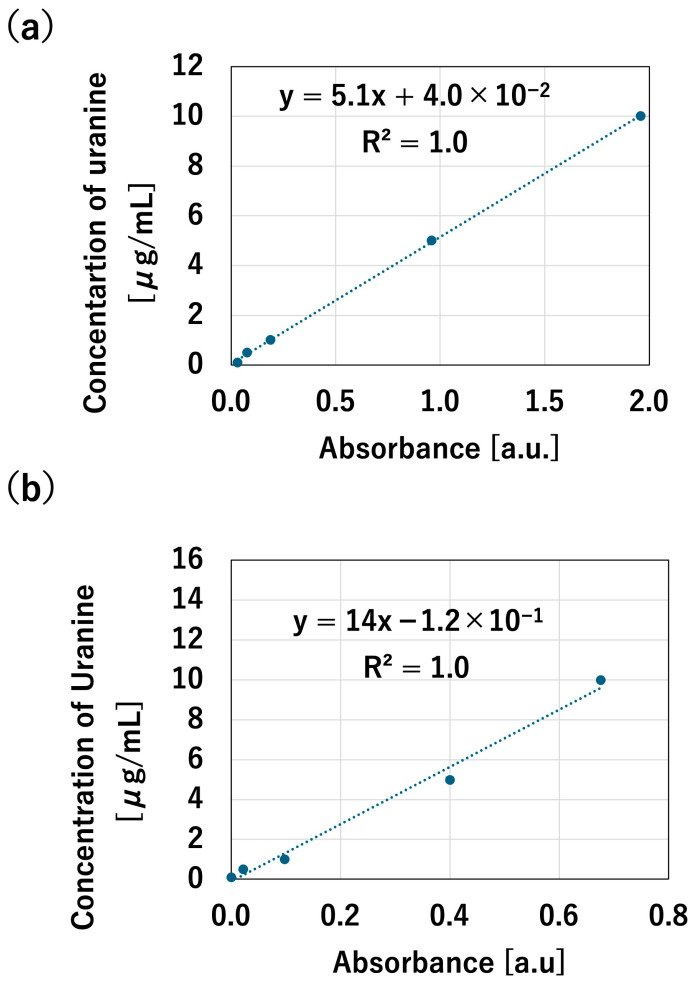
Calibration curve. (**a**) Uranine concentration in DPBS. (**b**) Uranine concentration in artificial sweat.

**Figure 7:**
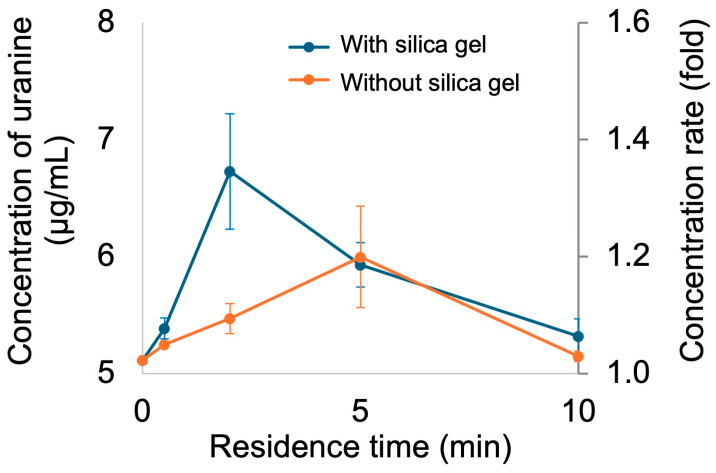
Effect of residence time on concentration rates in microchannel with/without packed silica gel at room temperature. The left vertical axis indicates the uranine concentration (µg/mL), which was calculated from the calibration curves shown in Figure 6. Data are presented as mean ± standard deviation (n = 3).

Next, the fundamental characteristics of the silica gel-filled microchannel were evaluated. A calibration curve was created to quantify the uranin concentration (Figure 6), and concentration tests were performed using a solution of uranin dissolved in DPBS (5 μg/mL). Investigation of the relationship between residence time in the channel and concentration rate revealed that, under room temperature conditions, the concentration rate increased as the residence time increased up to 2 min, with a maximum concentration of 1.4 times observed (Figure 7). However, the concentration ratio decreased at residence times of 5 and 10 min. This is thought to occur because an unconcentrated solution flows in after the silica gel reaches its moisture absorption limit, diluting the concentrated sample. Furthermore, a comparison with a microchannel that was not filled with silica gel showed that the silica gel-filled channel exhibited a higher concentration rate. This result suggests that filling with silica gel allows more efficient removal of solvent vapor from the liquid sample. In this study, silica gel was adopted as the desiccant because of its chemical stability, high availability, and well-established moisture absorption properties. Silica gel was selected in this study because of its moderate adsorption rate, chemical stability, and availability. Compared to molecular sieves, which exhibit higher adsorption capacity but require high-temperature regeneration, silica gel is more suitable for disposable or single-use microfluidic devices. Although calcium chloride is inexpensive, it tends to deliquesce, which may cause channel blockage in confined microstructures.

Table 1 summarizes the previously reported sample concentration approaches for sweat and aqueous biological samples. Many existing methods rely on solvent evaporation assisted by heating or complete drying of absorbent substrates, which limits their applicability to real-time or wearable systems. In contrast, this study demonstrates passive sample concentration at room temperature by combining the vapor permeability of PDMS with moisture adsorption by silica gel, achieving up to a 1.4-fold increase without external energy input. Although the concentration factor is moderate, the simplicity and power-free operation make this approach particularly suitable as a disposable pretreatment module for wearables.

In this study, uranine was employed as a model compound to isolate the effect of solvent removal from complex biological matrix effects. The use of real human sweat introduces significant variability owing to individual differences, contamination issues, and ethical constraints. Future studies should focus on validating this approach using actual sweat samples containing glucose and lactate.

**Figure 8:**
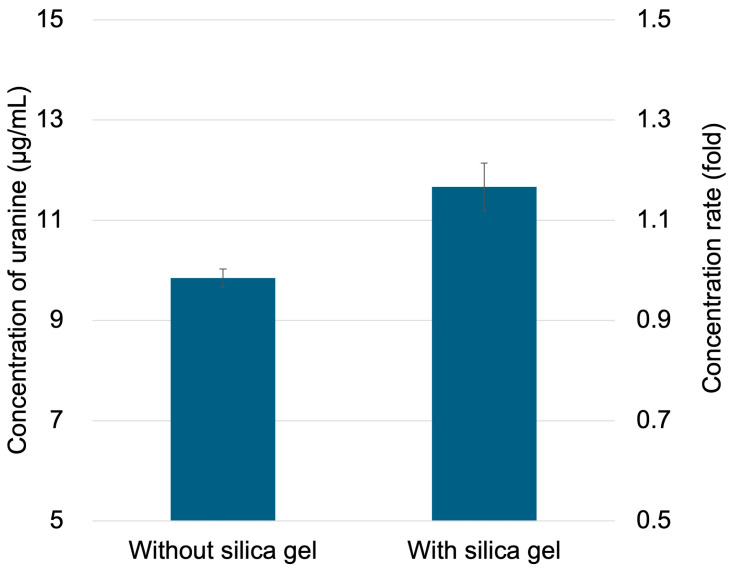
Concentrating uranine in artificial sweat using a microchannel with packed silica gel. Data are presented as mean ± standard deviation (n = 3).

Furthermore, concentration tests were performed using a solution of uranine dissolved in artificial sweat solution. Adopting the 2-min residence time that yielded the highest concentration rate, the uranine concentration increased 1.2-fold in the silica gel-filled microchannel (Figure 8). In contrast, no concentration was observed in the microchannel without the silica gel. These results demonstrate that this method enables the concentration of sweat components. Although the concentration factor achieved in this study is limited, the objective of this method is not a high-ratio concentration but rather its suitability for integration into existing devices as a simple pretreatment module requiring no external power source or heating.

Future studies should explore the control of hygroscopic behavior and optimal residence time using desiccants with different particle sizes and surface characteristics. Furthermore, optimizing the channel length and pillar structure can enable effective control of the residence time without altering the flow rate. Designed as a disposable pretreatment module, this device may not pose significant practical constraints for wearable applications, depending on its specific purpose.

The pillar spacing was designed to balance particle retention and flow resistance. Although parameter optimization was not within the scope of this study, the design framework allows for straightforward modifications in future studies.

## 4. Conclusions

In this study, we developed a PDMS-based microchannel packed with silica gel for the efficient concentration of sweat components. This microchannel demonstrated the ability to concentrate fluorescent dyes (sugar model) in sweat at room temperature without requiring an external power source or heating system. While this study evaluated the system using fluorescent dyes as a sugar model, verifying its applicability to diverse components found in actual sweat remains a challenge. The fabricated microchannel features a simple structure and does not require large external equipment, making it a potential pretreatment module for wearables. However, challenges remain in the implementation of wearable devices, including long-term skin adhesion, biofouling, and passive sweat transport. These aspects will be addressed by integrating skin-compatible adhesives and anti-fouling coatings into future designs. Generally, simple and flexible PDMS devices are well suited for skin attachment and integration with wearable devices [27]. This microchannel consists of a simple PDMS structure and is designed to be incorporated as a preprocessing module into existing wearable devices. A key feature of this method is its ability to improve sample conditions without modifying the detection unit itself, suggesting potential applications for concentrating low-molecular-weight components in sweat samples.

## Figures and Tables

**Table 1:** Comparison of previously reported sweat or aqueous sample concentration methods and their operational requirements.

Study	Target Sample/Analyte	Concentration Principle	External Energy	Concentration Factor	Remarks
Wong et al., 2014, Anal. Chem. [25]	Biological fluids (small metabolites)	Solvent evaporation on paper (heating)	Yes (heating)	Not reported quantitatively	Simple paper-based microfluidic device-based evaporation; not wearable
Hooton & Li, 2017, Anal. Chem. [26]	Human sweat metabolites	Passive drying on absorbent patch	No	Qualitative enrichment (not expressed as a single factor)	Passive drying-based enrichment (off-chip)
This study	Sweat model (uranine)	PDMS vapor permeation + silica gel adsorption	No	1.2–1.4×	Power-free, room temperature, wearable-compatible design

## Data Availability

The original contributions presented in this study are included in the article. Further inquiries can be directed to the corresponding author.
